# Treatment of Purulent Conjunctivitis

**Published:** 1893-03-25

**Authors:** 


### BRISTOL EYE HOSPITAL.

### Treatment of Purulent Conjunctivitis.

In the purulent conjunctivitis of the new-born babe, commonly known as Ophthalmia neonatorum, it is, unfortunately, by no means an unusual thing for several days to have elapsed since the commencement of the inflammation before the patient comes under the care of the oculist. Mothers are too ill, of course, to attend to the children, the midwife considers she has done her duty when she has applied cold tea or spring water, and it is not until the purulent discharge has fully manifested itself that some kind neighbour volunteers to bring the baby to the hospital. The infant is then from one to three weeks old, and the following history is given. About two or three days after birth the child's eyelids appeared puffy, and were stuck together by their margins, on which scabs formed. After bathing them with warm water they were able to open them, and some matter escaped, which in a few days became copious, and had to be constantly washed away. As fast as it was washed away the "corruption" worked out of the eyes again.

When a baby arrives at the clinic with such a history the nurse thoroughly cleanses the eyelids with an antiseptic solution, such as 1 in 5,000 of perchloride of mercury, or boracic acid lotion, and the child's head being placed between the knees of the surgeon, he first thoroughly inspects the cornea, separating the eyelids as widely as possible by means of retractors or the spring eye speculum; if the cornea is then found free from ulceration, and after the discharge is washed away appears bright and healthy looking, attention is given to the palpebral conjunctiva, the eyelids being everted, and all discharge wiped away with cotton wool; a solution of nitrate of silver of 10 grs. to the ounce is applied by means of a camel hair brush to the whole of the conjunctival surface that can be got at, after which a solution of common salt is sometimes applied, some surgeons thinking it advisable, others never using it unless a stronger solution of nitrate of silver be used. The person bringing the child is then instructed to wash the eyes out every hour, in bad cases night and day, and to apply an antiseptic lotion, the different surgeons favouring one more than another, boracic acid, boroglyceride, pyoktanin; creolin perchloride of mercury 1 in 5,000 is chiefly used by Mr. Cross, while Dr. Hailes has a preference for a 1 per cent. solution of sulphate of thallin. Bad cases have to attend daily and undergo careful inspection, and if considered advisable, the nitrate of silver solution is again applied, the surgeons always applying this themselves. As the eye shows signs of improvement, astringent lotions are used, sulphate of alum 4 grs. to the ounce, sulphate of zinc 1 to 2 grs. to the ounce, sulphate of copper 1 to 2 grs. to the ounce, being those most commonly used at this hospital.

When, however, after having removed the discharge from the cornea with dossils of lint, the cornea is found dull of appearance, like that of a dead fish, or with much chemosis, swelling around it, so that it appears sunk, and is in imminent danger from the cutting off of its blood supply by this swelling constricting the marginal vessels, attempts must be redoubled to subdue the inflammation and swelling, and hot fomentations are ordered, atropin drops instilled to dilate the iris, lest inflammation may spread to it, and it be bound down by plastic effusion, at the same time antiseptic washes being freely used. In other cases the cornea has gone on to ulceration, and great care needs to be exercised lest perforation occur and the eye be lost. Then every care is directed to the safety of the other eye. The ulceration is treated with quinine and eserin lotions in addition to the antiseptic washings.

When opacities have been left obscuring central vision, and no hope is entertained of the opacity diminishing, the eye is submitted to the operation for artificial pupil when the child is old enough.

The purulent conjunctivitis occurring in adults is happily much rarer, as it is much more severe in character, and is the result of infection with gonorrhœal discharge. As in the purulent conjunctivitis of the new-born child, the whole thickness of the conjunctiva is affected, and the specific inflammation attacks the subconjunctival tissues, placing the cornea, and with it the rest of the eye, in imminent danger. The same principles of treatment are carried out, and as one eye alone is generally affected the other eye is at once protected by means of a shield, introduced by Dr. Buller, consisting of a watch glass, the rim of which is imbedded between two rings of sticking plaster, the one attached to the convex side of the watch glass overlapping that attached to the concave side by about one-quarter of an inch, so that it can be applied all round the eye; a small space is left at the outer canthus for ventilation. The diseased eye is well irrigated with antiseptic lotions every hour, and the eyelids painted with a 2 per cent. solution of nitrate of silver if they can be everted. If the cornea is not affected ice pads and also hot fomentations are used to subdue the inflammation and swelling of the eyelids, and a ½ per cent. solution of nitrate of silver is dropped between the eyelids, after applying cocaine every hour till the discharge is diminished; it is then withheld until the discharge begins to increase again, antiseptic lotions of perchloride of mercury 1 in 5,000, or thallin sulphate 1 per cent., &c., being applied meanwhile. If there is much chemosis, and it yields not to ice and fomentations, small incisions are made in the conjunctiva, and bleeding encouraged, for which purpose leeches are also applied to the temple. An ointment of mercury, iodoform, iodol, boracic acid, or other antiseptic is applied to the lids. When the swelling is very excessive, so that the cornea cannot be seen, the outer canthus is divided with a pair of scissors or the knife, and when the conjunctiva is so chemosed around the cornea as to almost hide it, and to constrict the vessels supplying the cornea, removal of the overlapping fold relieves it. Astringents are used in the later stages after the acute symptoms have passed off—weak nitrate of silver, sulphate of zinc, or alum solutions. The inflammation may result in (1) recovery, in (2) recovery with opacity of the cornea, (3) sloughing of the cornea and loss of the lens and vitreous, (4) prolapse of the iris, (5) anterior staphyloma, (6) pyramidal cataract, which require their own appropriate treatment.

